# Antimutagenic and anticlastogenic effects of Turkish Black Tea on TA98 and TA100 strains of *Salmonella typhimurium* (*in vitro*) and mice (*in vivo*)

**DOI:** 10.1080/13880209.2017.1282969

**Published:** 2017-02-28

**Authors:** Mohammad Charehsaz, Hande Sipahi, Ashok Kumar Giri, Ahmet Aydin

**Affiliations:** Department of Toxicology-34755, Faculty of Pharmacy, Yeditepe University, Istanbul, Turkey

**Keywords:** Genotoxicity, chromosomal aberration assay, Ames test

## Abstract

**Context:** Black tea has been reported to have significant antimutagenic and anticarcinogenic properties associated with its polyphenols theaflavins (TF) and thearubigins (TR). Similarly, Turkish black tea (TBT) also contains a considerable amount of TF and TR.

**Objective:** This study investigated the mutagenic, antimutagenic and anticlastogenic properties of TBT.

**Materials and methods:** The mutagenic and antimutagenic effects of TBT (10 to 40000 μg/plate) were investigated *in vitro* on *Salmonella* strains TA98 and TA100 with and without S9 fraction. Anticlastogenic effect was studied at concentrations of 300–1200 mg/kg TBT extract by chromosomal aberrations (CA) assay from bone marrow of mice.

**Results:** The results of this study did not reveal any mutagenic properties of TBT. On the contrary, TBT extract exhibited antimutagenic activity at >1000 μg/plate concentrations in TA98 strain with and without S9 activation (40% inhibition with S9 and 27% without S9). In TA100 strain, the antimutagenic activity was observed at >20,000 μg/plate TBT extracts without S9 activation (28% inhibition) and at >1000 μg/plate with S9 activation (59% inhibition). A significant decrease in the percentage of aberrant cells (12.33% ± 1.27) was observed in dimethylbenz(*a*)anthracene (DMBA) plus highest concentration (1200 mg/kg) of TBT extract-treated group when compared to only DMBA-treated group (17.00% ± 2.28).

**Discussion and conclusion:** Results indicated that TBT can be considered as genotoxically safe, because it did not exert any mutagenic and clastogenic effects. As a result, TBT exhibited antimutagenic effects more apparently after metabolic activation in bacterial test system and had an anticlastogenic effect in mice.

## Introduction

Tea is one of the most consumed beverages around the world. It is prepared by the infusion of *Camellia sinensis* (L.) Kuntze (Theaceae) leaf (Coimbra et al. 2006). There are more than 300 types of tea leaves commercially available and mainly categorized into three groups according to the process used for the preparation of the tea leaves, i.e. green, oolong and black tea. Among all these different types of tea, fermented black tea constitutes 75% of the worldwide tea production (Sang et al. 2011). Different types of biological activities and beneficial health effects of tea have been reported in previous studies. Its role against cancer, cardiovascular diseases, congenital abnormalities, neurodegenerative diseases, depression, and many other diseases have been discussed in literature (Hayat et al. 2015). Recent epidemiological studies have reported the protective effects of black tea consumption against some types of cancer including ovarian (Baker et al. 2007), lung (Wang et al. 2014) and prostate (Fei et al. 2014) as well as reducing the risk of cardiovascular diseases (Arab et al. 2009; Grassi et al. 2015).

Protective measures against carcinogenic development in the body are currently one of the leading research areas. Genotoxic effects such as mutations and chromosomal aberrations (CA) are one of main causes of cancer (Valdiglesias et al. 2009). Hence, mutagenicity, antimutagenicity and CA have been studied by many research groups. Black tea has been reported to have antimutagenic and anticlastogenic properties (Zengin et al. 2014; Hayat et al. 2015). Although the mechanism of antimutagenic and anticarcinogenic activities of black tea has not yet been fully understood, it has been reported that these activities are due to the presence of its polyphenols theaflavins (TF) and thearubigins (TR) (Gupta et al. 2002; Bhattacharya et al. 2014). Henning et al. (2003) studied polyphenol contents of 11 black tea samples from different blends. Their TF contents were in the range of 7.3–20 mg/100 mL after the preparation of standard infusion methods. TF content of Turkish black tea (TBT) was found as 18 mg/g dry weight (Üstündağ et al. 2016). TBT was the focus of our study since the difference in theaflavin content can change the protective effect of tea.

Although there are some studies regarding the TBT ingredients (Turkmen et al. 2007; Alasalvar et al. 2013; Üstündağ et al. 2016) no evidence was found related to its antimutagenic, anticlastogenic or anticarcinogenic activities. Considering the extent of worldwide studies on these kind of activities of green and black tea, this study investigates the antimutagenic and anticlastogenic effects of TBT using *Salmonella* mutagenicity assay *in vitro* and clastogenicity assay *in vivo* in mice.

## Materials and methods

### Chemicals

Dimethylbenz(*a*)anthracene (DMBA), sodium azide (SA), 4-nitro-*o*-phenylenediamine (NPD), biotin, nicotinamide adenine dinucleotide phosphate (NADP), glucose-6-phosphate, ampicillin trihydrate, benzo(*a*)pyrene (B(*a*)P), colchicine and agar were supplied from Sigma Chemical Company. Histidine was provided from Fluka and nutrient broth was supplied from Hi Media laboratories Ltd.

### Animals

Male Balb C inbred mice, 12–14 weeks old, weighing 28–32 g, were used for the clastogenic and anticlastogenic assay and Sprague–Dawley male rats, weighing 150 to 200 g, were used for S9 preparation for this study. Animals were obtained from the Yeditepe University Medical School Experimental Research Center (Atasehir, Istanbul). They were housed in cages under standard condition and fed with standard rodent pellet diet and water *ad libitum* at 12 h light/dark cycle. Room temperature and relative humidity conditions were 28 ± 2 °C and 60% ± 5, respectively. The experimental protocol was approved by the Ethic Committee of Yeditepe University (No. 407).

### Bacterial strains

Mutagenicity and antimutagenicity assays were carried out using the *Salmonella* strains of TA98 and TA100. The TA98 strain was provided by Gazi University, Faculty of Pharmacy, Department of Toxicology (Ankara, Turkey) and the TA100 strain was received from the Marmara Research Center (Marmara Research Center of the Scientific and Technological Research Council of Turkey, Gebze, Turkey). TA98 detects frame shift mutations and TA100 detects base pair mutations. These strains were primarily recommended by Maron and Ames (1983) for routine mutagenicity assays.

### Preparation of S9 fraction

Rat liver homogenate was prepared according to the methods as explained by Ames et al. (1973), Garner et al. (1972) and Halder et al. (2005). Sprague–Dawley male rats, weighing 175–200 g, were fed with 0.1% phenobarbital in their drinking water for 7 days. On day 6, no food was supplied to the rats and they were euthanized on the following day. The rat liver homogenate (S9) was prepared by centrifugation at 9000 *g*, as explained by Maron and Ames (1983). Approximately 2 mL of S9 fractions were distributed in different small sterile cryovials, quickly frozen and stored at −80 °C for further use.

### Preparation of lyophilized TBT extract

TBT infusion was prepared by submerging 20 g of grindered TBT leaves (CAYKUR, Filiz Cayi) in boiling distilled water (500 mL) for 5 min. After 5 min of brewing, tea infusion was filtered immediately, frozen at −20 °C and then lyophilized (Christ, Alpha 2-4 LD, Germany). Approximately 2 g of lyophilized TBT extract was obtained from TBT infusion and stored at +4 °C until further analysis.

### Mutagenicity and antimutagenicity assays

For mutagenicity assay of TBT, standard plate incorporation test was carried as explained by Maron and Ames (1983). Both mutagenicity and antimutagenicity assays were performed with *Salmonella* tester TA98 and TA100 strains. TBT extract dissolved in hot distilled water and in different concentrations (0, 10, 100, 1000, 10000, 20000 and 40000 μg/plate) were used for both mutagenicity and antimutagenicity assay against the known mutagens and carcinogens. For testing mutagenicity, plates were co-incubated with the bacterial strains and prepared concentrations of TBT extract. Plates were inverted and placed at 37 °C for 48 h in dark, and revertant colonies were counted after incubation. To evaluate the impact of TBT metabolites, similar experiments were also carried out by incubating bacteria and extract with liver S9 fraction. Four plates were used for each concentration tested for experiments both with and without S9 fraction.

Antimutagenicity assays were carried out similarly using the same bacterial strains and the same TBT extract concentrations with and without S9 fraction. NPD was used as positive mutagen for TA98 strain without S9 and SA was used as positive mutagen for TA100 strain. B(*a*)P was used as positive mutagen for both TA98 and TA100 strains with S9 experiment. After incubation of inverted plates at 37 °C for 48 h in dark, revertant colonies were counted. The number of revertant colonies grown on plates containing the mutagen without TBT extract was defined as 100% which means 0% inhibition. The percentage of inhibition was calculated according to the formula: [(A−B)/(A−C)] × 100, where A = No. of histidine revertants in the absence of sample, B = No. of histidine revertants in the presence of sample, C = spontaneous revertants. The antimutagenic effect was considered moderate when the inhibitory effect was 25–40% and strong when it was more than 40%. Inhibitory effect less than 25% was considered as weak and was not recognized as a positive result (Zengin et al. 2014). Four plates were used for each TBT extract concentration for both with and without S9 experiments.

## *In vivo* CA assay

### Dose determination

Dose determination of TBT extract was based on the average daily tea consumption. It has been reported that approximately 5 cups (approximately 500–600 mL) of tea infusion per day is required to reduce the risk of some types of human cancer (Apostolides et al. 1996). Ground TBT leaves (20 g) brewed for 5 min in 500 mL of water equals to 5 cups of tea infusion per day for an adult. This means 2 g of TBT extract per day. In terms of body weight, this amount equals to 30 mg/kg daily for an adult weighing 65 kg. Considering this calculation, three dose levels at 300, 600 and 1200 mg/kg body weight were selected. These relatively higher doses were selected to justify the cumulative effects of tea consumption over a long period of time since our experimental protocol is based on only one application to animals.

### Assay protocol

For CA assay, three different concentrations (300, 600 and 1200 mg/kg body weight) of TBT extract were administered by gavage to 3 sets of 4 animals in each (a total of 12 mice). Immediately after administration of TBT extract, DMBA prepared in corn oil was administered at 50 mg/kg dose by gavage to all the 12 TBT-treated mice. Four mice were also gavaged with distilled water, treated with DMBA and served as positive control. Similarly 4 mice were gavaged with distilled water, treated with corn oil, and served as negative control. Twenty-two h after gavage, all animals were injected with colchicine and euthanized by cervical dislocation after 2 h. Chromosomes from bone marrow were prepared as explained by Gupta et al. (2002). Bone marrow is the target tissue in this test since it is a highly vascularized tissue and it contains a population of rapidly cycling cells that can be readily isolated and processed. All the slides were coded and 75 well spread metaphase cells (40 ± 2 chromosomes) per animal were scored for CA. A total of 300 metaphase cells were scored for each dose of TBT plus DMBA and for both negative, positive controls and only TBT-treated groups. For analysis of Mitotic Index (MI), 1000 cells/animal were scored and expressed in percentages. CA was scored as explained in the WHO (World Health Organization 1985) and OECD guidelines (Organization for Economic Co-operation and Development 1997). Frequencies of chromatid and chromosome-type aberrations per cell were calculated. Gaps were also recorded as explained in the OECD guideline but not included as percentage of aberrant cells or as the frequency of aberrations per cell.

### Statistical analysis

Experimental results were expressed as mean ± standard deviation. Dunnett and Crisafio (1995) multiple comparisons were carried out for mutagenicity and antimutagenicity data and for CA analysis data. *p* < 0.05 was considered as statistically significant.

## Results

In this study, the mutagenic and antimutagenic activities of TBT extract (with doses of 40,000 μg/plate and lower) were investigated. The results showed that in the presence of the different concentrations of TBT extract, the mutation frequencies for the tested *S. typhimurium* strains TA98 and TA100 did not change significantly when compared to spontaneous mutation frequencies. Any of the tested concentrations did not induce a significant increase in the revertant number of TA98 and TA100 strains with or without S9 activation. Also, the results revealed that the different concentrations of TBT extract did not influence the viability of bacterial strains. The average revertant colony numbers in negative control were 30.50 ± 3.87 for TA98 and 175.25 ± 18.64 for TA100 with S9 and 28.25 ± 6.08 and 173.50 ± 8.81 without S9, respectively. The plates with the positive control mutagens showed significant increases relative to the spontaneous mutation rate in the two tested strains.

The revertant colony numbers observed in the antimutagenicity assay and inhibitory rate percentages of the TBT extract with and without S9 activation were given in Table 1. A significant decrease in the revertant colonies were observed from the positive mutagen (NPD) plus 1000 μg/plate TBT extract in TA98 without S9 activation when compared to only positive mutagen treated plates. TBT extract exhibited strong antimutagenic activities at doses of 10,000, 20,000 and 40,000 μg (70%, 78%, and 80% inhibition, respectively) and moderate antimutagenic activity (27% inhibition) at the dose level of 1000 μg against NPD in the absence of S9 mix in *S. typhimurium* TA98.

**Table 1.** Antimutagenic activity of Turkish black tea (TBT) in *Salmonella* strains TA98 and TA100 with and without metabolic activation (S9) against known positive mutagens.

		TA98		TA100	
		Without S9		Number of revertant/plate	İnhibition (%)	Number of revertant/plate	İnhibition (%)
Negative control		28.25 ± 6.08		173.50 ± 8.81	
Positive control		629.75 ± 54.84		667.75 ± 47.40	
TBT Dose (μg/plate) + Positive mutagen (control)	40,000	149.75 ± 11.59*	80	433.75 ± 40.57*	47
	20,000	162.25 ± 12.82*	78	527.25 ± 62.60*	28
	10,000	210.25 ± 59.81*	70	606.25 ± 34.77	12
	1000	467.25 ± 58.03*	27	622.75 ± 39.45	9
	100	619.50 ± 28.24	2	643.50 ± 49.59	5
	10	613.75 ± 31.48	3	662.00 ± 50.65	1
With S9					
Negative control		30.50 ± 3.87		175.25 ± 18.64	
Positive control		84.25 ± 14.20		671.50 ± 34.89	
TBT Dose (μg/plate) + Positive mutagen (control)	40,000	29.25 ± 1.71*	97	180.00 ± 7.12*	99
	20,000	38.00 ± 8.37*	86	218.75 ± 38.80*	91
	10000	49.75 ± 12.50*	64	277.50 ± 58.25*	79
	1000	59.50 ± 10.28	40	381.00 ± 50.15*	59
	100	82.25 ± 15.59	4	631.75 ± 65.56	8
	10	84.00 ± 16.51	0.5	649.00 ± 53.81	5

* *p* < 0.05; Positive mutagen vs. positive mutagen plus Turkish black tea treatment group. Dunnett multiple comparisons test. Sterile distilled water (100 ml/plate) was used as negative control. 4-Nitro-*o*-phenylenediamine (NPD) (20 μg/plate) was used as positive control (mutagen) for *S. typhimurium* TA98 strain and Sodiumazide (SA) (1 μg/plate) was used as positive control (mutagen) for *S. typhimurium* TA100 without S9 activation. Benzo(*a*)pyrene (B(*a*)P) (1.0 μg plate) was used as positive control (mutagen) for both strains with S9 activation.

TBT extract showed strong antimutagenicity at the dose of 10,000 (64% inhibition), 20,000 (86% inhibition) and 40,000 μg (97% inhibition) against B(*a*)P; 1000 μg dose of the extract exhibited moderate (40% inhibition) antimutagenic activity in the presence of S9 mix in TA98 strain. On the other hand, 100 and 10 μg doses of the extract were found to be weak antimutagenic with and without S9 activation in this strain.

**Figure 1.**
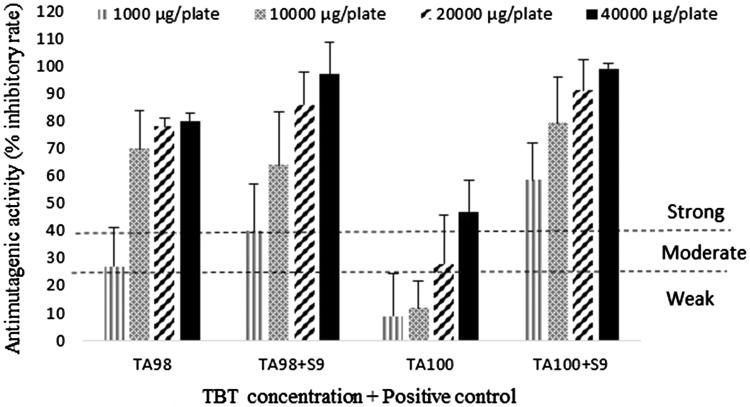
Antimutagenic activity (% inhibitory rate) of Turkish black tea extract against positive control (mutagen). 4-Nitro-o-phenylenediamine (NPD) (20 μg/plate) was used as positive control (mutagen) for *S. typhimurium* TA98 strain and sodium azide (SA) (1 μg/plate) was used as positive control (mutagen) for *S. typhimurium* TA100 without S9 activation. Benzo(*a*)pyrene (B(*a*)P) (1.0 μg plate) was used as positive control (mutagen) for both strains with S9 activation.

In TA100 strain, the antimutagenic effects were observed from the positive mutagen plus 20000 μg/plate TBT extract without S9 activation and from 1000 μg/plate with S9 activation. It was seen that TBT extract manifested moderate antimutagenicity (28% inhibition) at concentrations of 20000 μg and strong antimutagenic activity (47% inhibition) at dose of 40,000 μg against SA, while 10, 100, 1000, and 10,000 μg dose of extract were found to be weak antimutagenic in the absence of S9 mix in TA100 strain. On the other hand, except for 10 and 100 μg, all doses exhibited strong antimutagenic activity against B(*a*)P in the presence of metabolic activation system. The highest inhibition ratio (99%) was observed in 40,000 μg/plate dose group, followed by 20,000 μg (91%), 10000 μg (79%) and 1000 μg (59%) (Table 1, Figure 1).

Table 2 shows the antigenotoxic effects of TBT extract in bone marrow cells of mice against the known carcinogen DMBA. A decrease in the CA was observed in the three higher concentrations (300, 600 and 1200 mg/kg body weight) of TBT extract plus DMBA treated series when compared with the group treated with only DMBA. A statistically significant decrease in CA was observed only in the highest dose of TBT extract plus DMBA treated group when compared to only DMBA treated group. No significant changes were observed when CA induced by only 1200 mg/kg of TBT extract compared to negative control series.

**Table 2.** Chromosomal aberrations induced by DMBA and DMBA plus Turkish black tea (TBT) extract *in vivo* on bone marrow cells of mice.

Treatment (mg/kg)	Metaphase cells scored	Gaps^a^	Aberrations/cells^b^		Aberrant cells % (Mean ± S.D.)^c^	Mitotic indices (Mean ± S.D.)^c^
			Chromatid type	Chromosome type		
DMBA (50mg/kg)	300	31	0.127	0.073	17.00 ± 2.28	1.57 ± 0.24
TBT 300 + DMBA	300	28	0.117	0.037	14.67 ± 3.08	1.72 ± 0.29
TBT 600 + DMBA	300	25	0.127	0.047	15.67 ± 2.52	1.66 ± 0.16
TBT 1200 + DMBA	300	23	0.103	0.030	12.33 ± 1.27*	1.62 ± 0.25
Water control	300	11	0.133	0.040	4.35 ± 1.68	2.18 ± 0.27
Only TBT 1200	300	16	0.147	0.093	6.00 ± 1.68	1.93 ± 0.29

a Total chromatid and chromosome gaps at each dose were recorded but not included as aberrations/cell.

b Total number of aberrations (chromatid and chromosome type)/total number of cells scored per dose. Results are of 4 animals (75 metaphase cells/animal).

c Results at each dose were compared to those of control using Dunnett’s multiple comparison with control.

* *p* < 0.05 when compared with DMBA treated only. TBT 300 + DMBA:300 mg/kg TBT + DMBA; TBT 600 + DMBA:600 mg/kg TBT + DMBA; TBT 1200 + DMBA:1200 mg/kg TBT + DMBA; Only TBT 1200:1200 mg/kg TBT. DMBA: dimethylbenz(*a*)anthracene.

## Discussion

Previous reports have already shown that Indian black tea has significant antimutagenic and anticarcinogenic activities (Gupta et al. 2002; Halder et al. 2005, 2008; Bhattacharya et al. 2014). These reports indicated that the antimutagenic and anticancer activities of black tea are mainly due to the presence of TF and TR content of black tea.

According to our results TBT extract was neither toxic nor mutagenic to the bacteria at the tested concentrations, and bacterial growth was normal.

In the antimutagenicity assay of our study, the protective effect of TBT against the known mutagens in both TA98 and TA100 strains increased after metabolic activation with S9 (Figure 1) indicating the apparent protective effect of TBT metabolites against mutation in these strains. The effect of metabolic activation on the antimutagenic activity of plant polyphenols was also stated by Edenharder et al. (1997). Buening et al. (1981) have also postulated that some flavonoids such as TF and TR are potent inhibitors of cytochrome P450 monooxygenases such as CYP1A1 and CYP1A2. This inhibition prevents mutagenic/carcinogenic metabolite formation from some procarcinogenic chemicals such as B(*a*)P and aflatoxin B1.

Overall, our findings of decreased number of revertant colonies in both TA98 and TA100 with or without S9 activation clearly indicate that TBT extract has significant antimutagenic effects against the known mutagens.

According to our clastogenicity and anticlastogenicity data, TBT extract did not exert clastogenicity at a dosage of up to 1200 mg/kg *in vivo* in mice when compared to distilled water control. In anticlastogenic study, CA was decreased in all the three concentrations of TBT plus DMBA-treated series when compared with only DMBA-treated series, but statistically significant decrease in CA was observed in the highest dose (1200 mg/kg) of TBT plus DMBA-treated group when compared with only DMBA-treated group.

Previous studies revealed that the antimutagenic and anticlastogenic properties of tea polyphenols are mostly due to their antioxidant activity which inactivates direct carcinogens and inhibits the activation of indirect carcinogens extracellularly. Polyphenols also induce cytochrome P450 resulting in detoxifying of the carcinogens intracellularly (Gupta et al. 2002). TF play an important role in the antimutagenic activity of black tea by scavenging free radicals and also inhibiting cytochrome P450-dependent bioactivation of the carcinogens (Shiraki et al. 1994; Catteral et al. 1998). In the present study the anticlastogenic effect of TBT against the known mutagen DMBA is in agreement with the previous reports on the anticlastogenic effects of Indian black tea and its polyphenols TF and TR against the known mutagens DMBA and cyclophosphamide (CP) *in vivo* in mice (Gupta et al. 2002; Halder et al. 2005). Halder et al. (2005) studied anticlastogenic effect of TF and TR by CA and micronuclei formation (MN) method and reported that TF was more potent than TR in their *in vitro* test system. The TF content of TBT, 18 mg/g dry weight, is higher than those of 11 different blends of black tea samples which are between 7.3 and 20 mg/100 mL (Üstündağ et al. 2016; Henning et al. 2003). Henning et al. (2003) obtained their results by preparing tea bag infusions containing 1.5–2.4 g black tea leaves per bag in 100 mL hot water. We can assume that 2 g of dry TBT leaves give 38 mg TF after infusion with 100 mL hot water. Therefore, our significant antimutagenic and anticlastogenic effects may be mainly due to the presence of high-TF content in TBT extract. This is the first report of the antimutagenic and anticlastogenic effects of TBT *in vitro* in *Salmonella* assay and *in vivo* in mice. Further studies will confirm the antimutagenic and anticlastogenic effect of TBT due to its high-TF content.
